# Supplementary material for: Site-Specific Microbial Decomposer Communities Do Not Imply Faster Decomposition: Results from a Litter Transplantation Experiment
Source: Microorganisms. 2019 Sep 12;7(9):349. doi: 10.3390/microorganisms7090349 (PMC6780308; doi:10.3390/microorganisms7090349)
Supplement: Supplementary file 1 [file microorganisms-07-00349-s001.zip › bani_SM2019_reviewed.docx]

Supplementary material

Fig S1 Fungal trophic mode. Each box plot is the log10 transformed count of the relative guild. A) Total count across all the samples of the experiment, B) fungal guild relative to the Monticolo samples, C) fungal guild relative to Renon and D) fungal guild relative to San Genesio Atesino. Pa = Pathogen, Sa = Saprotroph, Sy = Symbiotroph, Pat = Pathotroph, U = Unassigned

Fig S2 Class composition. A) Bacterial class composition and B) fungal class composition. Each panel represents a different site of decomposition: M Monticolo, RN Renon and SG San Genesio Atesino

Fig S3 A) Quantification of 16S DNA marker gene and B) Quantification of ITS marker gene. Each boxplot represent three samples. Each panel represents a different of decomposition: M Monticolo, RN Renon and SG San Genesio Atesino. Different letters indicate significant difference as function of time effect (*p*-value < 0.05, ANOVA followed by HSD test).

Fig S4 Chemical composition of oak litter and mass loss. A) Mass loss described as percent, B) C/N ratio, C) percent of C of the litter and D) percent of N content of the litter. Values are mean vales ± standard deviation (n=3). Different colours are the site of decomposition: M Monticolo, RN Renon and SG San Genesio Atesino.

Fig S5 Richness of fungal community. A) Total fungal community, B) Saprotrophic fungal community. Each boxplot represent three samples. Each panel represents a different site of decomposition: M Monticolo, RN Renon and SG San Genesio Atesino

Fig S6 Co-occurence between Bacteria (x axis) and Saprotrophic Fungi (y axis). Only statistically significant correlation are shown (Spearman test, *p*-value < 0.05). Red color is for negative co-occurence while blue is for positive co-occurence.

Fig S7 Co-occurence between Bacteria (x axis) and Fungi (y axis). Only statistically significant correlation are shown (Spearman test, *p*-value < 0.05). Red color is for negative co-occurence while blue is for positive co-occurence.

Table S1. K-rate and mass remaining express as percent. Values are mean vales ± standard deviation (n=3). M Monticolo, RN Renon and SG San Genesio Atesino. No statistical difference found between the different sites.

|  | k-rate | Mass remaining % |
| --- | --- | --- |
| M | 0.61 ± 0.05 | 40.62 ± 9.1 |
| RN | 0.57 ± 0.1 | 44.99 ± 6.46 |
| SG | 0.65 ± 0.18 | 42.39 ±3.16 |

Table S2. GLMs applied on the total fungal community. For each model, the AIC score and if the factor was statistically significant is provided. Models with * include the interaction between the factor, models including + instead not account for interaction between the different factors. Best fitting models are highlighted in bold. p < 0.001 ***, p <0.005 **

| Model | Variables | AIC  Score Fungal community |
| --- | --- | --- |
| Null | 1 | 32928 |
| Model 1 | Time*** | 31023 |
| Model 2 | Site*** | 32272 |
| Model 3 | Total N*** | 31587 |
| Model 5 | C/N ratio*** | 31657 |
| Model 7 | Time***+Site*** | **30065** |
| Model 8 | Time***×Site** (Time×Site)*** | 30387 |

Table S3. p-value of ANOVA on bacterial genus composition for the different GLM models.

| bacterial genus | time | site | mass | N |
| --- | --- | --- | --- | --- |
| Acidicapsa | 0.001 | 1 | 0.8 | 1 |
| Acidisoma | 0.001 | 0.114 | 1 | 0.999 |
| Actinokineospora | 0.001 | 0.974 | 0.998 | 0.34 |
| Actinomycetospora | 0.01 | 0.001 | 1 | 0.999 |
| Actinoplanes | 0.001 | 0.469 | 1 | 0.049 |
| Aeromicrobium | 0.184 | 0.95 | 0.992 | 1 |
| Amycolatopsis | 0.001 | 0.815 | 1 | 0.254 |
| Aquisphaera | 0.001 | 0.009 | 1 | 1 |
| Armatimonadetes_gp5 | 0.417 | 0.001 | 1 | 1 |
| Asticcacaulis | 0.001 | 1 | 0.627 | 0.001 |
| Bdellovibrio | 0.001 | 0.95 | 1 | 0.982 |
| Beijerinckia | 0.184 | 0.853 | 1 | 1 |
| Blastocatella | 0.001 | 1 | 1 | 1 |
| Brevundimonas | 0.184 | 1 | 1 | 0.074 |
| Burkholderia | 0.001 | 0.975 | 1 | 1 |
| Caedibacter | 0.001 | 1 | 1 | 1 |
| Caulobacter | 0.001 | 1 | 0.999 | 0.428 |
| Chitinophaga | 0.001 | 1 | 1 | 0.977 |
| Chlorophyta | 0.001 | 0.214 | 0.999 | 1 |
| Chryseobacterium | 0.184 | 0.998 | 1 | 0.123 |
| Conexibacter | 0.001 | 0.991 | 1 | 0.685 |
| Cryptosporangium | 0.001 | 0.017 | 1 | 0.999 |
| Cystobacter | 0.076 | 0.537 | 1 | 1 |
| Deinococcus | 0.001 | 0.964 | 1 | 1 |
| Devosia | 0.001 | 1 | 1 | 0.458 |
| Dokdonella | 0.001 | 0.043 | 0.705 | 0.999 |
| Dongia | 0.001 | 1 | 1 | 0.999 |
| Duganella | 0.097 | 1 | 1 | 0.138 |
| Dyadobacter | 0.656 | 1 | 1 | 0.027 |
| Edaphobacter | 0.001 | 0.972 | 0.989 | 0.47 |
| Epilithonimonas | 0.093 | 1 | 0.873 | 0.91 |
| Ferruginibacter | 0.001 | 0.037 | 1 | 1 |
| Flavobacterium | 0.072 | 1 | 1 | 0.641 |
| Fluviicola | 0.586 | 0.853 | 1 | 0.878 |
| Friedmanniella | 0.001 | 0.077 | 1 | 1 |
| Gemmata | 0.001 | 1 | 1 | 0.044 |
| Gemmatimonas | 0.001 | 1 | 1 | 0.712 |
| Geodermatophilus | 0.001 | 0.609 | 1 | 0.999 |
| Gp3 | 0.001 | 1 | 1 | 0.999 |
| Gp4 | 0.001 | 1 | 1 | 0.747 |
| Granulicella | 0.001 | 0.917 | 1 | 1 |
| Hymenobacter | 0.001 | 0.9 | 1 | 0.093 |
| Ilumatobacter | 0.001 | 1 | 1 | 0.91 |
| Jahnella | 0.001 | 0.998 | 0.168 | 1 |
| Kineococcus | 0.001 | 0.986 | 1 | 1 |
| Kineosporia | 0.008 | 0.421 | 1 | 1 |
| Labrys | 0.001 | 1 | 0.809 | 0.999 |
| Legionella | 0.384 | 0.974 | 1 | 1 |
| Luteibacter | 0.001 | 1 | 1 | 0.99 |
| Luteolibacter | 0.001 | 1 | 1 | 0.148 |
| Marmoricola | 0.189 | 0.012 | 1 | 1 |
| Massilia | 0.001 | 0.974 | 1 | 1 |
| Methylobacterium | 0.024 | 0.005 | 0.999 | 1 |
| Methylorosula | 0.001 | 0.974 | 1 | 1 |
| Mucilaginibacter | 0.001 | 0.276 | 0.42 | 1 |
| Mycobacterium | 0.001 | 0.681 | 0.995 | 0.976 |
| Nakamurella | 0.053 | 1 | 1 | 1 |
| Nannocystis | 0.001 | 1 | 0.999 | 0.365 |
| Naxibacter | 0.231 | 1 | 1 | 0.998 |
| Niastella | 0.12 | 0.001 | 1 | 0.001 |
| Nocardioides | 0.368 | 0.598 | 1 | 1 |
| Novosphingobium | 0.51 | 1 | 1 | 0.977 |
| Ohtaekwangia | 0.001 | 0.998 | 1 | 1 |
| Opitutus | 0.001 | 1 | 0.975 | 1 |
| Patulibacter | 0.006 | 1 | 1 | 1 |
| Pedobacter | 0.586 | 1 | 1 | 0.401 |
| Peredibacter | 0.517 | 1 | 1 | 1 |
| Phaselicystis | 0.001 | 1 | 0.453 | 0.999 |
| Phenylobacterium | 0.001 | 1 | 0.934 | 0.712 |
| Polaromonas | 0.04 | 0.974 | 1 | 0.997 |
| Prosthecobacter | 0.012 | 1 | 1 | 1 |
| Pseudomonas | 0.656 | 0.999 | 1 | 0.854 |
| Pseudonocardia | 0.001 | 1 | 0.627 | 1 |
| Pseudoxanthomonas | 0.586 | 1 | 1 | 0.969 |
| Quadrisphaera | 0.001 | 0.917 | 1 | 0.955 |
| Rathayibacter | 0.01 | 1 | 1 | 1 |
| Rhizobacter | 0.384 | 1 | 1 | 1 |
| Rhizobium | 0.008 | 1 | 0.998 | 1 |
| Rhodanobacter | 0.004 | 0.031 | 1 | 1 |
| Rhodococcus | 0.092 | 1 | 1 | 0.91 |
| Rhodopseudomonas | 0.001 | 1 | 0.085 | 0.281 |
| Rickettsia | 0.667 | 0.041 | 0.992 | 1 |
| Roseomonas | 0.008 | 0.077 | 1 | 0.922 |
| Saccharibacteria_genera_incertae_sedis | 0.092 | 0.986 | 1 | 0.96 |
| Sediminibacterium | 0.001 | 1 | 1 | 1 |
| Silvimonas | 0.188 | 0.007 | 1 | 1 |
| Solirubrobacter | 0.001 | 0.987 | 1 | 0.614 |
| Spartobacteria_genera_incertae_sedis | 0.001 | 1 | 1 | 0.997 |
| Sphingobium | 0.004 | 1 | 0.314 | 0.628 |
| Sphingomonas | 0.189 | 0.127 | 1 | 1 |
| Spirosoma | 0.001 | 1 | 1 | 1 |
| Steroidobacter | 0.001 | 1 | 0.999 | 0.08 |
| Streptomyces | 0.002 | 0.045 | 0.998 | 0.977 |
| Streptophyta | 0.001 | 0.95 | 0.9 | 1 |
| Subdivision3_genera_incertae_sedis | 0.001 | 0.615 | 0.999 | 0.918 |
| Subtercola | 0.586 | 0.97 | 1 | 1 |
| Terriglobus | 0.001 | 0.998 | 1 | 0.977 |
| Terrimonas | 0.001 | 1 | 0.987 | 0.877 |
| Vampirovibrio | 0.001 | 0.974 | 1 | 1 |
| Variovorax | 0.021 | 1 | 0.9 | 0.999 |
| Verrucomicrobium | 0.001 | 1 | 0.995 | 0.51 |
| WPS.1_genera_incertae_sedis | 0.001 | 0.415 | 0.979 | 1 |
| WPS.2_genera_incertae_sedis | 0.001 | 0.271 | 1 | 1 |

Table S4. p-value of ANOVA on saprotrophic genus composition for the different GLM models.

| saprothropic genus | time | site | mass | C/N |
| --- | --- | --- | --- | --- |
| Alatospora | 0.001 | 0.001 | 1 | 1 |
| Alternaria | 0.001 | 1 | 0.043 | 0.363 |
| Amyloxenasma | 0.026 | 0.847 | 0.98 | 1 |
| Angustimassarina | 1 | 1 | 1 | 1 |
| Anthostomella | 0.998 | 0.389 | 1 | 1 |
| Apiotrichum | 0.896 | 1 | 1 | 1 |
| Apodus | 0.001 | 1 | 1 | 1 |
| Arachnopeziza | 0.189 | 0.001 | 1 | 0.998 |
| Arthrobotrys | 0.21 | 0.609 | 1 | 1 |
| Arthrocatena | 0.998 | 0.978 | 1 | 0.971 |
| Articulospora | 0.684 | 1 | 0.992 | 0.81 |
| Athelia | 1 | 1 | 1 | 1 |
| Auricularia | 0.028 | 1 | 1 | 1 |
| Bannoa | 0.001 | 0.031 | 1 | 1 |
| Brachyphoris | 0.318 | 1 | 1 | 1 |
| Calycina | 0.996 | 0.993 | 1 | 1 |
| Ceramothyrium | 1 | 1 | 0.994 | 0.998 |
| Chaetosphaeria | 1 | 1 | 1 | 1 |
| Chalara | 1 | 0.003 | 1 | 0.989 |
| Cladophialophora | 0.001 | 1 | 1 | 0.31 |
| Clavatospora | 0.001 | 0.998 | 1 | 0.391 |
| Clitocybe | 0.995 | 0.973 | 0.992 | 0.98 |
| Clitopilus | 1 | 1 | 1 | 1 |
| Clypeosphaeria | 1 | 1 | 1 | 1 |
| Coleophoma | 0.999 | 1 | 0.097 | 0.999 |
| Comoclathris | 0.177 | 1 | 0.964 | 0.966 |
| Coniothyrium | 0.001 | 1 | 1 | 1 |
| Cristinia | 0.799 | 0.998 | 1 | 1 |
| Crocicreas | 0.993 | 1 | 1 | 1 |
| Cystobasidiopsis | 0.287 | 0.966 | 1 | 1 |
| Cytospora | 0.944 | 1 | 1 | 1 |
| Dactylella | 0.001 | 1 | 1 | 1 |
| Dicarpella | 0.001 | 0.959 | 1 | 1 |
| Dictyochaeta | 1 | 1 | 1 | 1 |
| Dictyosporium | 0.904 | 1 | 1 | 1 |
| Didymella | 0.125 | 1 | 1 | 1 |
| Dinemasporium | 1 | 1 | 0.516 | 0.998 |
| Endoconidioma | 0.001 | 0.001 | 1 | 0.169 |
| Endosporium | 0.001 | 0.001 | 1 | 0.938 |
| Entoleuca | 0.833 | 0.001 | 1 | 0.994 |
| Entoloma | 0.939 | 1 | 0.027 | 1 |
| Eucasphaeria | 1 | 1 | 1 | 1 |
| Exophiala | 0.015 | 1 | 1 | 1 |
| Fenestella | 1 | 1 | 1 | 1 |
| Filobasidium | 0.001 | 1 | 1 | 1 |
| Flagelloscypha | 1 | 0.957 | 1 | 1 |
| Fusarium | 0.939 | 1 | 1 | 1 |
| Fusidium | 1 | 1 | 1 | 0.866 |
| Hansfordia | 0.042 | 1 | 0.961 | 0.988 |
| Helicoma | 1 | 1 | 1 | 1 |
| Herpotrichia | 0.042 | 1 | 1 | 1 |
| Heyderia | 1 | 1 | 1 | 1 |
| Hohenbuehelia | 0.094 | 1 | 1 | 1 |
| Hormonema | 0.904 | 1 | 1 | 1 |
| Hyalorbilia | 0.998 | 1 | 1 | 1 |
| Hymenoscyphus | 0.162 | 1 | 1 | 1 |
| Hypholoma | 0.858 | 0.99 | 1 | 1 |
| Incrucipulum | 0.939 | 1 | 1 | 0.998 |
| Infundichalara | 1 | 1 | 1 | 1 |
| Knufia | 1 | 0.055 | 1 | 0.995 |
| Lachnella | 1 | 1 | 1 | 1 |
| Lachnellula | 1 | 1 | 1 | 0.97 |
| Lachnum | 0.001 | 0.455 | 0.919 | 1 |
| Lanzia | 1 | 1 | 1 | 0.996 |
| Lasionectria | 1 | 1 | 1 | 1 |
| Lemonniera | 0.001 | 0.361 | 1 | 0.04 |
| Lophiostoma | 0.577 | 1 | 1 | 1 |
| Lophiotrema | 0.699 | 1 | 1 | 1 |
| Lophium | 1 | 1 | 1 | 1 |
| Meliniomyces | 0.901 | 1 | 0.034 | 1 |
| Menispora | 0.779 | 1 | 1 | 1 |
| Moellerodiscus | 1 | 1 | 1 | 1 |
| Montagnula | 0.994 | 1 | 1 | 1 |
| Mortierella | 0.286 | 1 | 1 | 1 |
| Mucor | 1 | 1 | 1 | 1 |
| Mycena | 0.001 | 1 | 0.129 | 0.998 |
| Mycetinis | 1 | 1 | 1 | 1 |
| Mycoarthris | 0.089 | 1 | 1 | 0.616 |
| Myrmecridium | 1 | 1 | 1 | 1 |
| Myrothecium | 1 | 1 | 1 | 1 |
| Nectria | 1 | 1 | 1 | 1 |
| Nectriopsis | 1 | 1 | 1 | 1 |
| Neosetophoma | 0.775 | 1 | 1 | 1 |
| Nigrospora | 1 | 1 | 1 | 1 |
| Ochroconis | 0.001 | 1 | 1 | 1 |
| Oliveonia | 0.252 | 0.001 | 1 | 0.996 |
| Orbilia | 1 | 0.078 | 1 | 1 |
| Paraconiothyrium | 1 | 1 | 1 | 1 |
| Parasola | 1 | 1 | 1 | 0.995 |
| Peniophora | 1 | 1 | 1 | 0.001 |
| Periconia | 0.745 | 1 | 1 | 1 |
| Pezicula | 0.858 | 1 | 1 | 1 |
| Pezizella | 0.684 | 0.351 | 1 | 1 |
| Phaeococcomyces | 0.001 | 0.97 | 1 | 0.719 |
| Phaeohelotium | 0.004 | 1 | 1 | 1 |
| Phaeosphaeria | 1 | 0.76 | 1 | 1 |
| Phaeosphaeriopsis | 0.001 | 1 | 1 | 1 |
| Phanerochaete | 0.993 | 1 | 1 | 1 |
| Phlebiella | 0.939 | 0.473 | 1 | 1 |
| Phoma | 0.001 | 1 | 0.981 | 1 |
| Plectania | 0.085 | 1 | 1 | 0.998 |
| Plenodomus | 0.125 | 1 | 1 | 1 |
| Podospora | 0.939 | 1 | 0.043 | 1 |
| Polydesmia | 1 | 1 | 1 | 1 |
| Pseudoplectania | 1 | 1 | 1 | 1 |
| Pyrenochaeta | 0.001 | 1 | 1 | 1 |
| Rhinocladiella | 1 | 1 | 1 | 1 |
| Rhizophlyctis | 1 | 1 | 1 | 1 |
| Rhodotorula | 0.001 | 1 | 1 | 1 |
| Roridomyces | 1 | 1 | 0.24 | 1 |
| Rosellinia | 1 | 0.278 | 1 | 1 |
| Rutstroemia | 0.998 | 0.985 | 1 | 1 |
| Saccharicola | 1 | 1 | 1 | 1 |
| Sarocladium | 0.672 | 1 | 1 | 1 |
| Scleropezicula | 0.684 | 0.692 | 1 | 1 |
| Sclerostagonospora | 0.026 | 1 | 1 | 1 |
| Sistotrema | 1 | 0.959 | 1 | 0.998 |
| Spirosphaera | 0.001 | 1 | 1 | 0.046 |
| Sporothrix | 1 | 1 | 0.293 | 0.994 |
| Stilbella | 0.806 | 1 | 1 | 1 |
| Subulicystidium | 0.004 | 0.001 | 1 | 1 |
| Sympodiella | 0.001 | 1 | 1 | 0.998 |
| Talaromyces | 1 | 1 | 1 | 1 |
| Teichospora | 0.999 | 1 | 1 | 1 |
| Tephrocybe | 1 | 1 | 1 | 1 |
| Tetracladium | 0.745 | 1 | 1 | 1 |
| Trechispora | 0.939 | 1 | 0.134 | 1 |
| Umbelopsis | 0.252 | 0.229 | 1 | 1 |
| Uncobasidium | 0.999 | 1 | 1 | 1 |
| Vermiculariopsiella | 0.9 | 0.803 | 1 | 1 |
| Xenasma | 0.112 | 1 | 1 | 1 |
| Xenopolyscytalum | 0.271 | 1 | 0.974 | 1 |
| Xylaria | 1 | 0.02 | 1 | 1 |
| Zalerion | 1 | 1 | 1 | 1 |
