# Supplementary figures and images for: Site-Specific Microbial Decomposer Communities Do Not Imply Faster Decomposition: Results from a Litter Transplantation Experiment
Source: Microorganisms. 2019 Sep 12;7(9):349. doi: 10.3390/microorganisms7090349 (PMC6780308; doi:10.3390/microorganisms7090349)

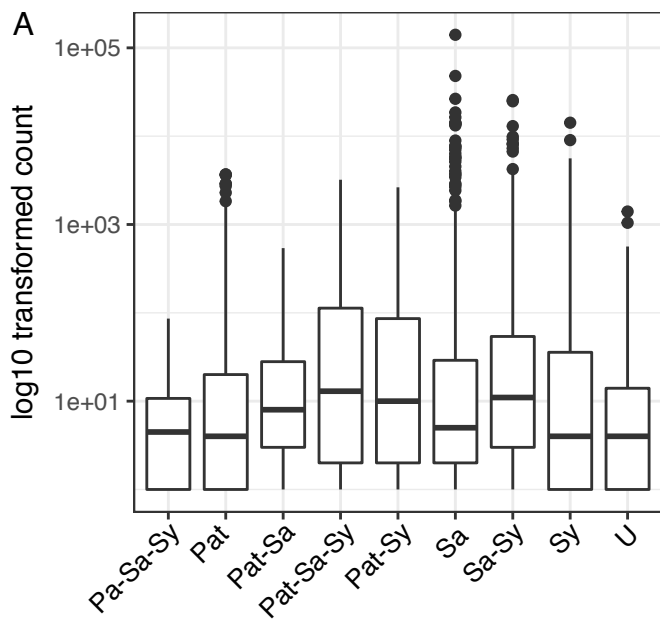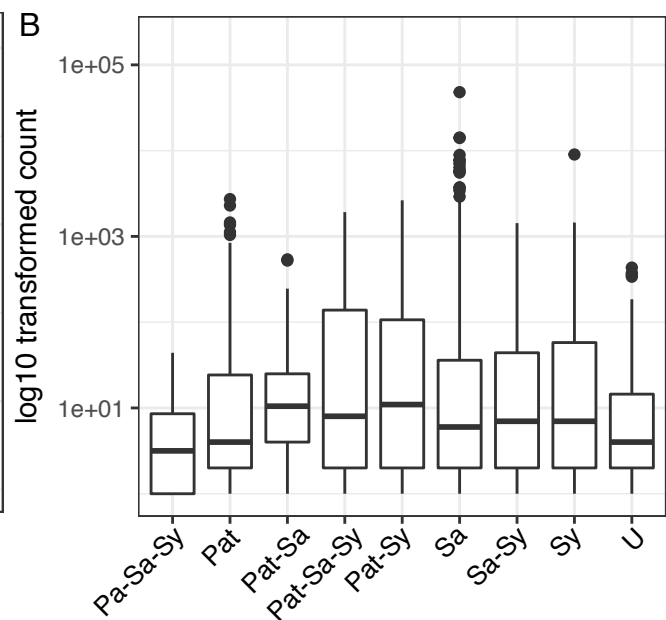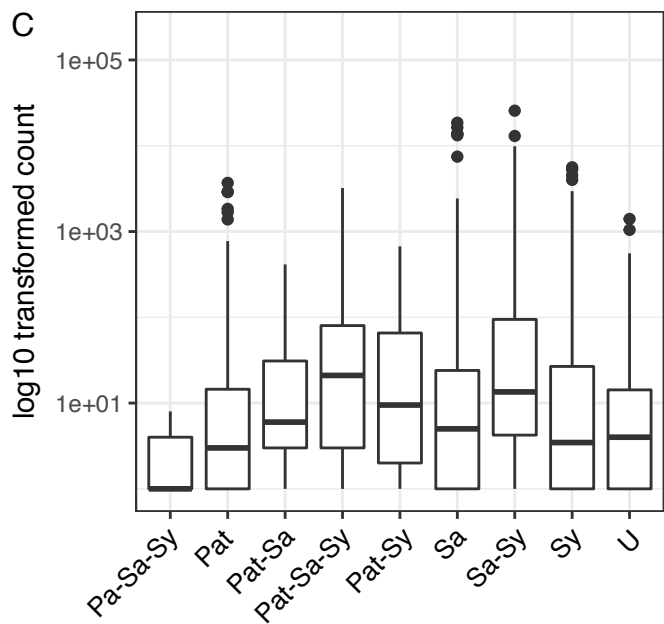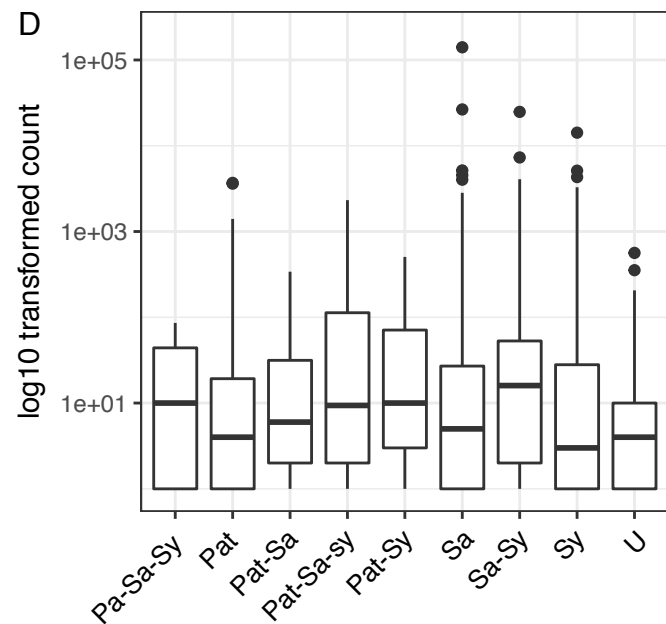

Supplement: Supplementary file 1 [file microorganisms-07-00349-s001.zip › figure_S1.pdf]

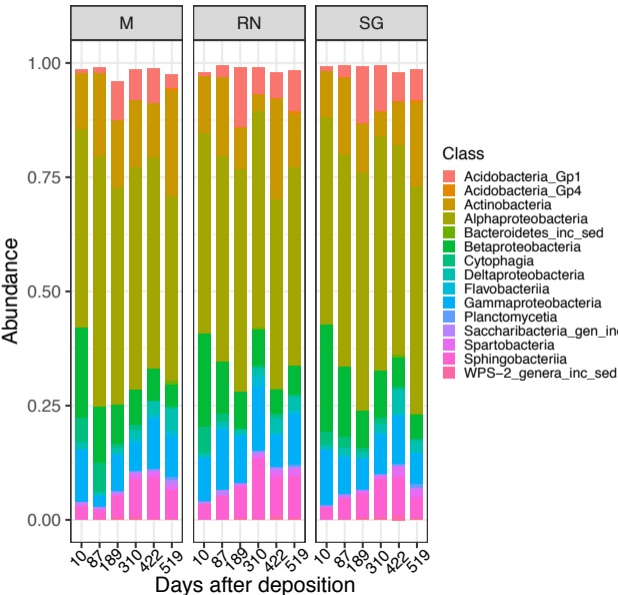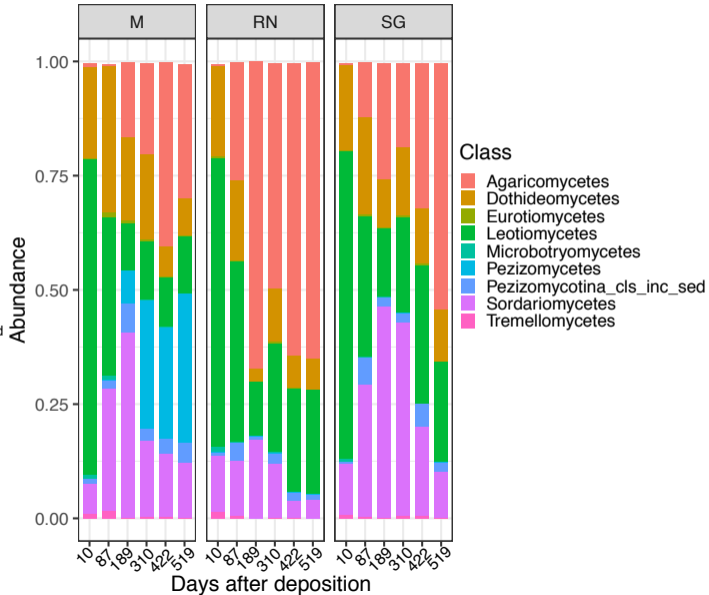

Supplement: Supplementary file 1 [file microorganisms-07-00349-s001.zip › figure_S2.pdf]

**A**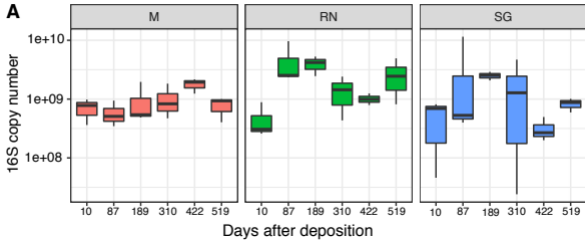**B**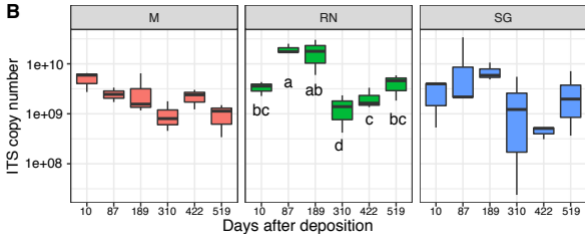

Supplement: Supplementary file 1 [file microorganisms-07-00349-s001.zip › figure_S3.pdf]

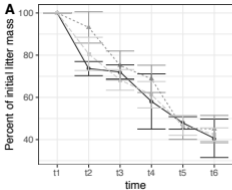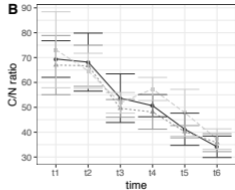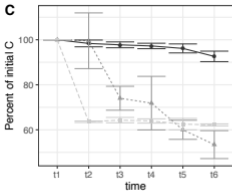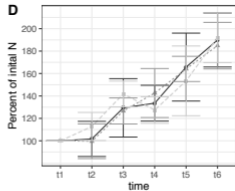

Days after deposition

Supplement: Supplementary file 1 [file microorganisms-07-00349-s001.zip › figure_S4.pdf]

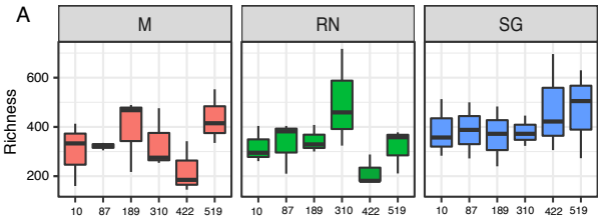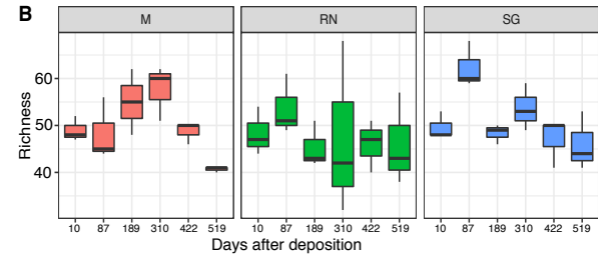

Supplement: Supplementary file 1 [file microorganisms-07-00349-s001.zip › figure_S5.pdf]

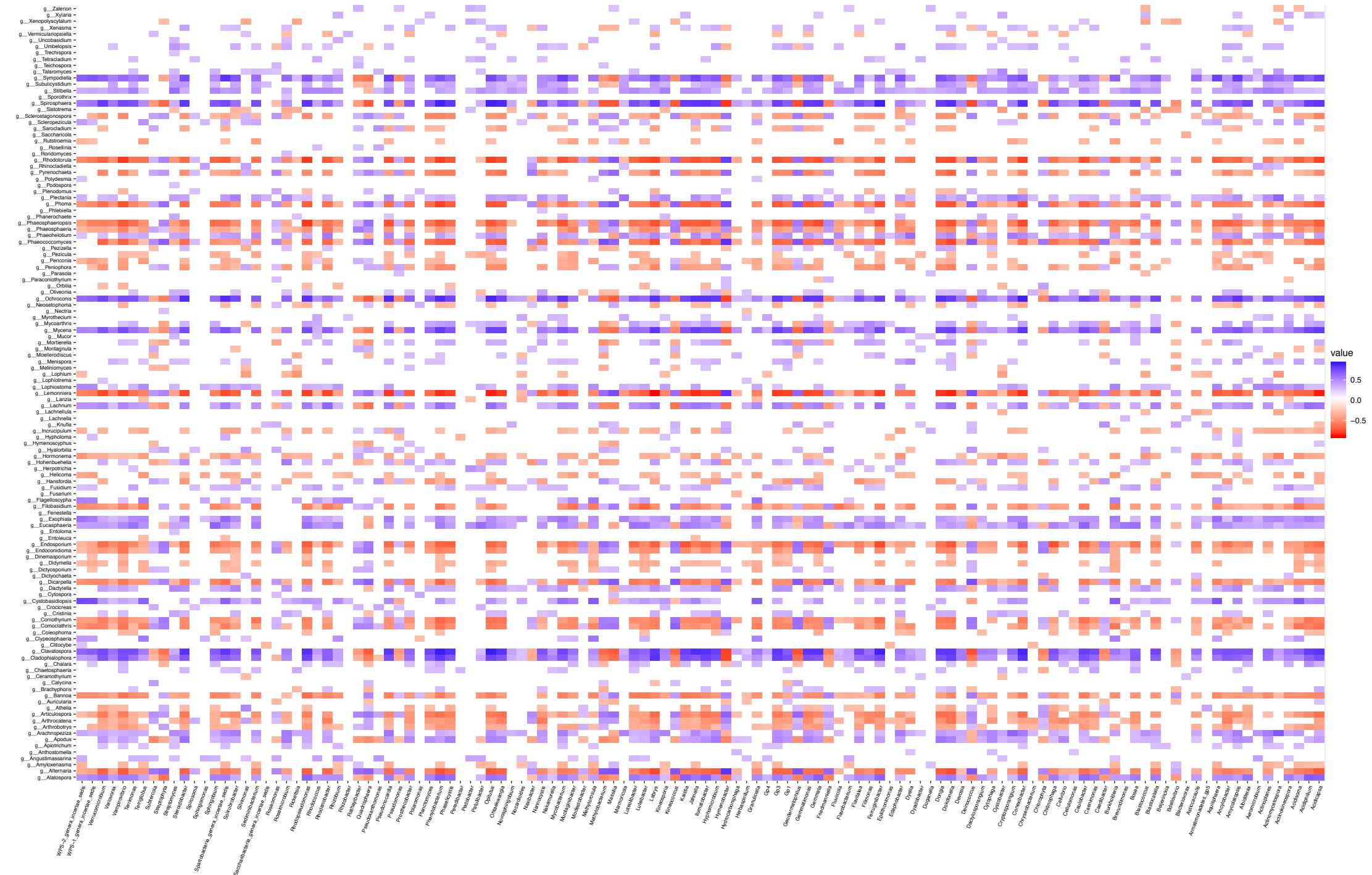

Supplement: Supplementary file 1 [file microorganisms-07-00349-s001.zip › Figure_S6.pdf]

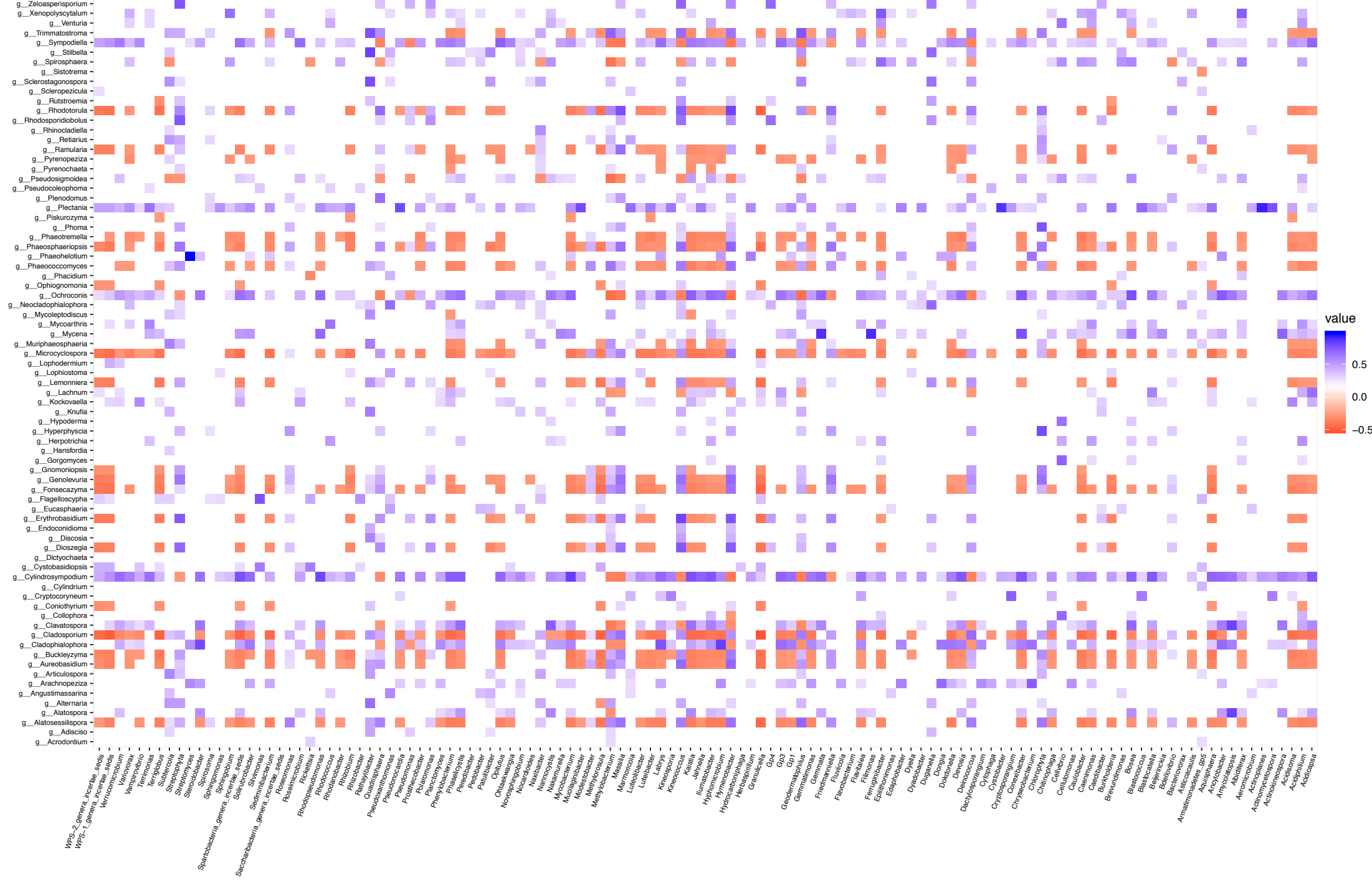

Supplement: Supplementary file 1 [file microorganisms-07-00349-s001.zip › FIgure_S7.pdf]
